# Optimizing outcomes with less than more: multi-institutional experience of fast-forward fractionation and the impact of dosimetric parameters on toxicity in breast cancer patients

**DOI:** 10.3389/fonc.2026.1861707

**Published:** 2026-06-10

**Authors:** H. Habibullah, M. Alotaibi, H. Almarzouki, Z. Mulla, M. Elbaiomy, R. Alahmadi, A. Ahmed, Y. Bahadur, R. Ujaimi, E. Senan, M. Attar, A. Bamakhramah, H. Altoukhi, H. Muamenah, O. Iskanderani, R. Garout, J. Alturkistani, H. Hijazi, Z. Jastaniah

**Affiliations:** 1 Department of Oncology, King Faisal Specialist Hospital and Research Centre, Jeddah, Saudi Arabia; 2 Department of Radiology, Faculty of Medicine, King Abdulaziz University, Jeddah, Saudi Arabia; 3 Medical Oncology Unit, Oncology Center, Mansoura University, Madinah, Egypt; 4 Department of Oncology, King Salman Bin Abdulaziz Medical City, Medinah, Saudi Arabia; 5 Department of Clinical Oncology, Sohag University Hospital, Sohag, Egypt; 6 Department of Internal Medicine, Faculty of Medicine, King Abdulaziz University, Rabigh, Saudi Arabia

**Keywords:** adjuvant radiotherapy, breast cancer, fast-forward, ultra-hypofractionation, toxicity

## Abstract

**Background:**

The FAST-Forward trial demonstrated the non-inferiority of 26 Gy in five fractions compared to 40 Gy in 15 fractions over five years. This study aimed to report observed breast toxicities and examine the impact of dosimetric parameters in ultra-hypofractionated radiotherapy (RT) across a diverse patient population in Saudi Arabia.

**Methods:**

This multi-institutional retrospective study evaluated 100 breast cancer patients treated with whole-breast adjuvant radiotherapy (26 Gy in 5 fractions) between June 2020 and June 2023, with adjuvant RT at two different institutions in Saudi Arabia. Breast toxicity data were retrospectively collected from medical chart review and verbal reports from patients lost to follow-up. Correlations between dosimetric factors, body mass index (BMI), and acute and late toxicity symptoms were statistically assessed.

**Results:**

A total of 85 patients were included in the analysis after excluding those with incomplete toxicity data with a median follow-up of 19 months. Breast skin dermatitis was the most common side effect (82.35%, n=70) with 5% of patients experiencing Grade III, followed by hyperpigmentation (72.9%, n=62), breast discomfort (30.3%, n=26) and breast hardness (17.6%, n=15). Breast pain rated ≥5 out of 10 on the pain scale was reported in 15% of cases. Breast pain had a significant correlation with larger breast volume and higher V107% values (p=0.013, and p=0.012 respectively). Furthermore, breast skin dermatitis was significantly correlated with breast CTV, BMI, and breast separation (p=0.0001, 0.015, and 0.001 respectively).

**Conclusions:**

Ultra-hypofractionated RT with a 26 Gy in five fractions schedule was well tolerated in our patient cohort. A larger breast volume, higher BMI, and increased V105% and V107% were associated with greater toxicity. Optimizing modifiable dosimetric factors may reduce adverse effects. Consequently, these findings offer valuable insights into treatment refinement.

## 1 Introduction

The incidence of breast cancer in Saudi Arabia has risen sharply, with a 186% increase from 783 cases diagnosed in 2004 to 2,240 cases in 2016 (1). Between 2009 and 2016, 43% of cases occurred among women aged 45–59 years and 30% among those aged 30–44 years (1). This increase was largely attributed to improved healthcare access, enhanced screening initiatives, and early detection programs (2). Socioeconomic and lifestyle changes have further contributed (2). A notable proportion of these cases was seen in younger women compared to Western populations (3), explained by a combination of genetic, environmental, and lifestyle factors (4).

Despite the rising incidence, advancements in early breast cancer management, particularly in radiotherapy [RT], have led to improved outcomes and reduced mortality worldwide. A meta-analysis of 78 randomized trials involving 42 thousand women showed that adjuvant RT after oncologic surgery in early-stage breast cancer resulted in a 19% absolute reduction in locoregional recurrence at 5 years and a 5% reduction in breast cancer deaths at 15 years (5). In Saudi Arabia, adjuvant RT have significantly improved prognosis, reduced local recurrence and enhanced disease-free survival (6). Patients treated with three-dimensional conformal RT showed better local control and survival outcomes.

Historically, the international standard for adjuvant RT before 2001 was 50 Gy in 25 fractions over five weeks (7, 8). This regimen aimed to maximize tumor control while minimizing late tissue toxicity using smaller daily doses. Interest in hypofractionated RT emerged after 2002, with larger doses delivered over fewer sessions (9). Trials such as START A and START B confirmed that 40–42.5 Gy over 15–16 fractions across 3 weeks were non-inferior to conventional schedules, also demonstrating reduced toxicity (9, 10). Consequently, hypofractionation has become the new standard of care globally. Recent landmark studies such as the FAST-Forward trials has advanced this further toward ultra-hypofractionation. Delivering 26 Gy in five fractions over one week proved non-inferior to 40 Gy over three weeks for local tumor control, with comparable normal tissue safety up to five years (11, 12). Both trials confirmed that fewer, larger fractions can maintain efficacy. As a result, the 5-fraction, 1-week schedule has been increasingly adopted globally. Although successful outcomes and comparative measures were evident, side effects and toxicity levels remained an area of particular interest. The toxicity profiles between hypofractionated and ultra-hypofractionated schedules became a point of clinical interest. Although the incidence of moderate or marked normal tissue effects was slightly higher in the 26 Gy group (11%) than the 40 Gy group [9%], the difference was not statistically significant (11). Globally, comparisons of toxicity between hypofractionation and ultra-hypofractionation remain limited, particularly in Middle Eastern populations (13, 14).

Our current study aims to address this gap by reporting outcomes, toxicity profiles, and the impact of dosimetric parameters of ultra-hypofractionated radiotherapy in Saudi women with early breast cancer, representing the first study of its kind in the region.

## 2 Methodology

### 2.1 Patients stratification: inclusion and exclusion

This multi-institutional retrospective study included 100 breast cancer patients treated with adjuvant radiotherapy between June 2020 and June 2023. All patients received 26 Gy in five fractions at King Faisal Specialist Hospital & Research Centre [KFSHRC] and King Abdulaziz University Hospital [KAUH) in Saudi Arabia. The study was approved by the biomedical ethics committees of both institutions [KFSHRC IRB#2023-53; KAUH Ha-02-J008]. Written informed consent was waived due to the retrospective nature of the study and minimal risk to patients. For patients lost to follow-up, additional data were sought via telephone contact, and verbal consent was obtained prior to data collection. Eligible participants were women aged ≥ 18 years with pathologically confirmed invasive carcinoma or ductal carcinoma *in situ* [DCIS] of the breast, clinical stage M0 [no distant metastasis], and who had undergone complete microscopic excision of the primary tumor through breast-conserving surgery, with or without reconstruction.

### 2.2 Radiotherapy protocol

All patients received whole breast RT only, with breast clinical target volume (CTV) delineation and contouring of organs at risk (ipsilateral lung, spinal cord, and heart for left-sided cases). Radiotherapy was forward-planned using 3D conformal radiotherapy (3D-CRT), with delivery via a segmental (step-and-shoot) technique, and an optional tumor bed boost. Planning followed dose constraints established in the FAST-Forward trial protocol, and patients who did not meet the dose constraints were treated with an alternative dose schedule (11). Standard 3D-CRT field borders were used for treatment planning, with the whole breast contoured as the clinical target volume (CTV), and treatment fields generated with a 7 mm margin from the CTV (15).

Acute and late toxicities were evaluated through a retrospective review of medical records, supplemented by structured telephonic assessments for patients lost to follow-up to ensure data continuity. Radiation dermatitis was graded according to the Radiation Therapy Oncology Group (RTOG) scale, while breast pain was quantified using the Numeric Pain Rating Scale (NPRS), an 11-point tool ranging from 0 (‘no pain’) to 10 (‘worst possible pain’) (16). Other adverse events, specifically breast edema, hyperpigmentation, induration, and change in breast shape, were captured via clinician-led assessment based on Common Terminology Criteria for Adverse Events (CTCAE v5.0) and patient-reported symptomatic complaints. To ensure assessment uniformity and comparability across both participating institutions, a unified data collection form was utilized for all clinical and telephonic evaluations. Acute toxicities were monitored and documented during treatment and within 3 months post-completion, while late toxicities were recorded during subsequent scheduled follow-up consultations, every 3 months.

Data included patient demographics, disease and treatment characteristics, RT dose-volume histogram parameters, treatment side effects, and disease outcomes. The study assessed correlations between independent variables, such as CTV volume, boost dose, Dmax, V107%, V105%, breast separation, Body Mass Index (BMI), chemotherapy history, and age, as well as dependent variables, including acute (Breast pain, overall pain, breast discomfort and radiation dermatitis) and late toxicities (skin pigmentation, change in breast shape, breast induration, and breast shape alterations), detailed in **Supplementary Material 1**.

### 2.3 Statistical analysis

Statistical analysis involved descriptive statistics and multivariate regression analyses; logistic regression was used for binary outcomes and linear regression for continuous outcomes. Statistical significance was set at p < 0.05. Data were analyzed using SPSS, Version 27.0 for Windows [SPSS Inc., Chicago, IL, USA].

## 3 Results

The patients’ ages ranged from 31 to 82 years, with a median age of 56 years. Comorbidities, including diabetes, hypertension, lung disease, prior malignancies, heart disease, asthma, osteoarthritis, hypothyroidism, and sarcoidosis, were present in 59 patients. All patients were diagnosed with breast cancer of various histological subtypes: invasive ductal carcinoma (IDC) (75%), invasive lobular carcinoma (10%), mixed carcinoma (1%), mucinous carcinoma (6%), desmoplastic carcinoma (1%), and papillary carcinoma (3%) (**Table 1**). Tumor grading varied from Grade I (24%) to Grade II (52%) and Grade III (21%), while ductal carcinoma *in situ* (DCIS) accounted for 5% of cases. Staging details are provided in **Table 1**. Lymphovascular invasion (LVI) was identified in 11% of patients. Although 46% of the patients were left-sided breast cancer cases, Deep Inspiration Breath Hold (DIBH) was not used in this study due to limited resources, increased workload and time constraints.

**Table 1:** Patient demographic data (n=100).

Characteristic	Distribution
Age	31–82 years (Median 56 years)
Laterality	Right breast (54%) Left breast (46%)
Stage	pTis (5%) pT1(40%) pT2(33%) pT3(1%) ypTis (2%) ypT1(6%) ypT2(5%) ypT3(1%) N0(99%) Nmic (1%) M0(100%)
Histological subtypes	IDC (75%) ILC (10%) Mixed (1%) Mucinous (6%) DCIS (5%) Desmoplastic (1%) Papillary (3%)
Receptor status	ER/PR +ve (68%) Triple –ve (17%) HER-2 +ve (7%) Triple + (5%)
Grade	GIII (21%) GII (52%) GI (24%)
LVI	11% +ve 74% –ve
Systemic therapy	Neoadjuvant chemotherapy (25%) Adjuvant chemotherapy (25%) Adjuvant hormonal therapy (74%)

*T, Tumor; N, Node; M, Metastasis; TiS, Tumor in Situ; Nmic, Micrometastasis in Nodes; IDC, Invasive Ductal Carcinoma; ILC, Invasive Lobular Carcinoma; DCIS, Ductal Carcinoma in Situ; Er, Estrogen Receptor, PR, Progesterone Receptor; G, Grading; LVI, Lymphovascular Invasion.

All patients underwent breast-conserving surgery with complete microscopic excision, followed by adjuvant RT. Among them, 89 underwent sentinel lymph node biopsy, 8 underwent axillary lymph node dissection, and 3 with DCIS did not require axillary surgery. Following surgery, estrogen receptor (ER) and progesterone receptor (PR) positivity was found in 68% of patients, HER2 positivity in 7%, and triple-negative breast cancer in 17%. Among the 100 patients, 25% received neoadjuvant chemotherapy, and 74% received adjuvant hormonal therapy (**Table 1**).

### 3.1 Impact of patient and dosimetric parameters on toxicity

All patients completed their planned RT without discontinuation owing to toxicity. The median follow-up period was 19 months [Range: 6–44 months].

Analysis of dosimetric parameters demonstrated high overall compliance with the FAST-Forward trial protocol constraints for organs at risk. Dose–volume histogram (DVH) evaluation was performed for all treatment plans to assess adherence to organ-at-risk constraints for the heart and ipsilateral lung across both institutions. The maximum reported dose (Dmax) was 108%, with no patients exceeding this constraint. For cardiac structures, the highest reported dose to 1.5Gy was 23%, and the maximum reported Heart V7 was 5.5%, with only 4 patients (4.7%) exceeding the 5% constraint. Regarding breast dosimetry, the highest reported V107% was 0.8%, and 5 patients (5.9%) exceeded the 5% breast V105% constraint. For the ipsilateral lung, the maximum Lung V8 was 17.4%, with 7 patients (8.2%) exceeding the 17% constraint.

Each plan was peer reviewed during the weekly Simulation Quality Assurance meeting, attended by multidisciplinary teams from both institutions, to ensure treatment plan quality and adherence to planning standards. Although minor deviations from predefined constraints were observed in a limited number of cases, the overall dosimetric results demonstrated robust compliance with protocol-defined organ-at-risk limits while maintaining appropriate target coverage.

**Figure 1:**
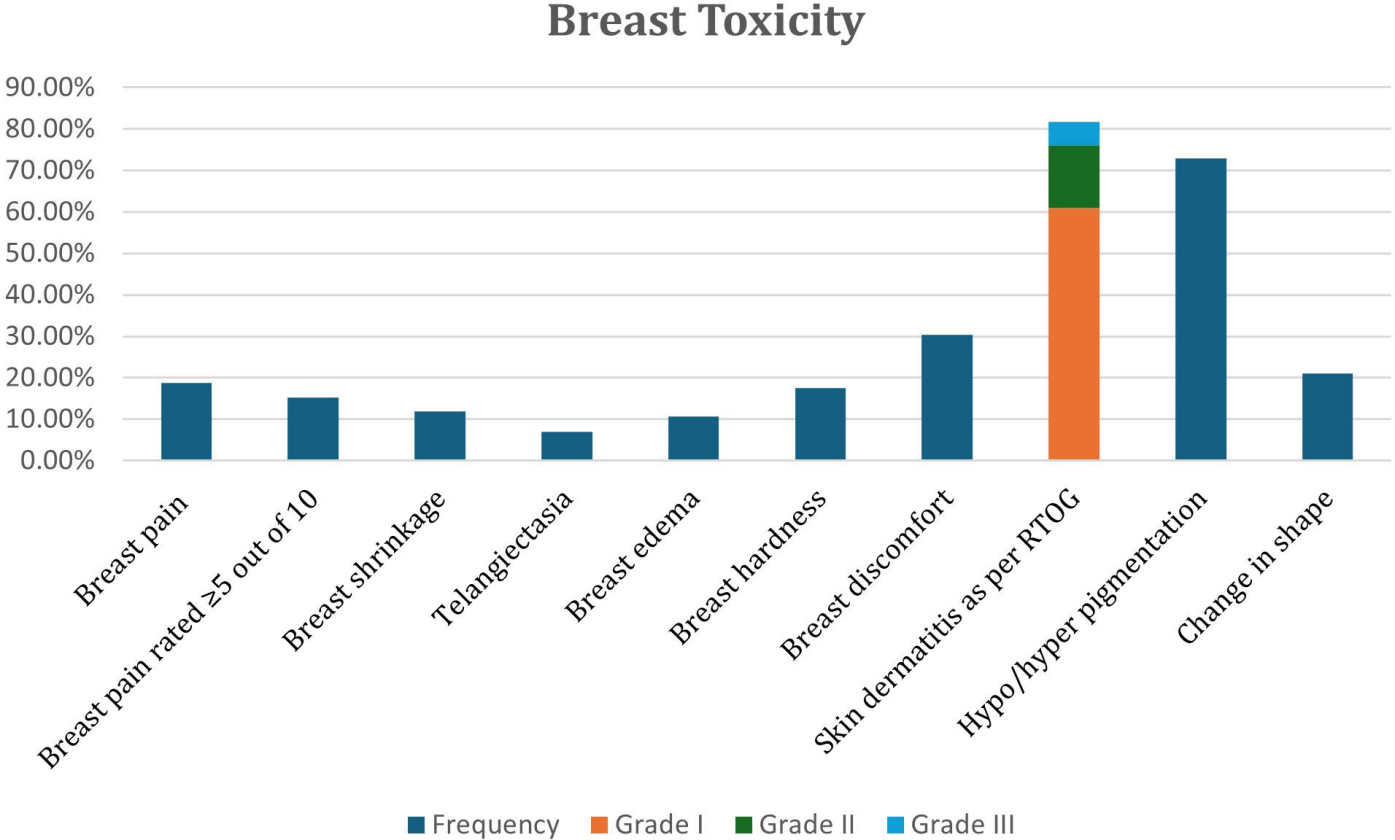
Percentage of patient-reported toxicities after radiotherapy (RT).

Treatment-related side effects were manageable and did not compromise the completion of therapy. A total of 85 patients were included in the toxicity analysis after excluding those with incomplete toxicity data. The frequencies of patient-reported toxicities are listed in **Table 2** and **Figure 1**. Breast skin dermatitis, as per the radiation therapy oncology group, was the most common side effect (82.35%, n=70), followed by hyperpigmentation (72.9%, n=62), breast discomfort (30.3%, n=26) and breast induration (17.6%, n=15). Breast pain rated above 5 on NPRS and overall reported pain (15.2%, n=13; 33%, n=28) was significantly correlated with larger breast CTV and higher V107% values (p=0.013 and p=0.012, respectively) (**Table 3**). Breast skin dermatitis was significantly associated with the breast CTV, BMI, and breast separation (p=0.0001, p=0.015, and p=0.001, respectively). Breast edema was significantly correlated with BMI and V105% (p=0.045 and p=0.037, respectively), while changes in breast shape were also associated with V105% (p=0.037) (**Table 3**). No significant correlations were observed between breast toxicity and the boost dose, plan Dmax, age, chemotherapy, or patient comorbidities.

**Table 2:** Frequencies of patient-reported toxicities after radiotherapy (RT) (n=85).

Type of Toxicity	Side effects	Frequency (n)%
Acute side effects	Breast pain (NPRS ≥5/10) Overall pain	(13) 15.2% (28) 33%
	Breast discomfort	(26) 30.3%
	Skin dermatitis (RTOG) Grade I Grade II Grade III	(52) 61.0% (13) 15.0% (5) 5.8%
	Breast edema	(9) 10.6%
Late side effects	Skin pigmentation (Hypo/hyper)	(62) 72.9%
	Change in breast shape	(18) 21%
	Breast hardness (Induration)	(15) 17.6%
	Breast shrinkage (Atrophy)	(10) 11.8%
	Telangiectasia	(6) 7.1%

NPRS, Numeric Pain Rating Scale; RTOG, Radiation Therapy Oncology Group; G, Grading.

**Table 3:** The relationship between patient-reported toxicity and BMI, breast CTV, V107 or 105 and separation (n=85).

Breast pain			
Variables	R (correlation coefficient)	F-statistic	p. value
Breast CTV in cc	.267	6.374	.013
V107%	.273	6.666	.012
Breast dermatitis			
	R (correlation coefficient)	F-statistic	p. value
BMI	.293	6.287	.015
Breast CTV in cc	.486	26.036	.0001
Separation	.350	11.724	.001
Breast edema			
	Coefficient (B)	Odds ratio	p. value
BMI	.544	1.723	.048			
V105%	1.057	2.879	.037
Change in breast shape			
	Coefficient (B)	Odds ratio	p. value
V105%	.363	1.438	.037				

CTV, Clinical Target Volume; V, volume of Tissue; BMI, Body Mass Index.

## 4 Discussion

Radiotherapy (RT) remains a cornerstone for improving local control and survival in patients with breast cancer, however, it is associated with a range of acute and late toxicities that can affect cosmetic outcomes and quality of life. Understanding the factors influencing radiation-induced toxicity is crucial for optimizing treatment planning and minimizing adverse effects while maintaining therapeutic effectiveness. Anatomical and dosimetric factors such as BMI, breast CTV, and breast separation are key predictors of radiation toxicity, as documented in previous studies (17–19).

Our findings were aligned with Wang et al., who reported that the acute toxicity profile of ultra-hypofractionated whole-breast irradiation is generally common but minimal, with good cosmetic outcomes in most cases (13). Krug et al. further noted that although ultra-hypofractionation offers logistical advantages, certain late toxicities might increase over time; however, the overall differences in toxicity remain modest, supporting the use of the 26 Gy regimen as a safe and effective alternative (14). In our analysis, a larger breast CTV and higher V107% were significantly associated with increased breast pain, while breast skin dermatitis correlated strongly with higher BMI, larger breast CTV, and greater breast separation (**Table 3**). These findings are consistent with prior research emphasizing dose-volume effects in moderately hypofractionated regimens (13, 20). Similar associations have been described in other studies, emphasizing the need to moderate high-dose volumes and fractions to control adverse effects (11, 13). Smith et al. also highlighted that, with moderate hypofractionation, patients should not be excluded based on anatomical characteristics if dose homogeneity and reduced V105% are achieved (21). Supporting this, minimizing dose heterogeneity significantly reduce breast skin dermatitis, even in patients with larger breast volumes (22).

The FAST-Forward trial (Brunt et al.) established that a five-fraction, one-week schedule yields lower rates of grade ≥3 acute skin toxicity than conventional fractionation, validating the safety of ultra-hypofractionated regimens (12). Our findings further indicate that breast edema is significantly linked to higher BMI and elevated V105%, while alterations in breast shape correlate specifically with increased V105% (**Table 3**).

Since larger doses per fraction amplify the biological impact of dose heterogeneity (23), optimizing treatment plans to minimize V105% and V107% is critical. Ultimately, evidence suggests that when dose homogeneity is strictly maintained, breast volume, rather than the fractionation schedule itself, becomes the primary driver of cosmetic morbidity (23). Setting stricter dosimetric thresholds could enhance treatment tolerability without compromising oncologic outcomes. Although formal cosmetic assessments were not systematically performed, the low rates of significant breast shrinkage and shape change suggest generally favorable cosmetic results. Overall, our findings support the safety and efficacy of the FAST-Forward schedule within our population and highlight actionable dosimetric considerations for personalized and safer RT planning.

The low incidence of severe breast skin dermatitis and pain in our cohort emphasize the feasibility and tolerability of this regimen. Shortened treatment courses offer significant benefits, improving patient quality of life and reducing hospital visits, which is particularly advantageous in the Saudi Arabian healthcare context (24). Economic and logistical advantages of hypofractionated schedules have also been emphasized in other studies as well, promoting broader applicability (20). Given the rising incidence of breast cancer in Saudi Arabia, such regimens have the potential to improve treatment accessibility and adherence, as demonstrated locally by Rudat et al. (25). Although the toxicity levels observed in our study were comparable to those reported in other cohorts (26), minor differences in skin toxicity suggest potential roles for genetic and physiological variations. Similar observations have been reported in Middle Eastern populations, reinforcing the importance of region-specific research to optimize RT protocols for different ethnic groups (27).

Overall, our findings contribute to the expanding evidence base supporting the use of ultra-hypofractionated RT as a new standard of care for appropriately selected patients with early-stage breast cancer, offering the potential to enhance access, reduce healthcare system burdens, and sustain excellent clinical outcomes globally. Future studies with larger patient cohorts are needed to validate these results and better assess long-term toxicity and disease control.

### 4.1 Clinical implications

The strong correlation between higher BMI, large breast volume, and toxicity suggests that anatomy-based dose escalation is the primary challenge in our region. For “high-risk” anatomy (high BMI or large CTV), optimizing dose homogeneity to stay within the safe thresholds established by the FAST-Forward trial, must be a priority. The implementation a “red-flag” system where any plan exceeding specific V105% or V107% thresholds undergo a mandatory peer review to ensure all optimization efforts have been exhausted before treatment. Greater adoption of advanced techniques, such as inverse planning IMRT, may improve dose homogeneity and limit normal tissue exposure, thereby minimizing toxicity in high-risk patients (11). Patients with left-sided breast cancer should be treated using a 3D-CRT technique incorporating DIBH whenever feasible to maintain mandatory cardiac dose constraints (28). Although most observed toxicities were mild to moderate, their clinical relevance remains important, as even low-grade toxicities can affect patient-reported outcomes, cosmetic satisfaction, and long-term breast quality. The incorporation of PROMs is crucial for future research.

### 4.2 Limitations

The study has several limitations. The relatively short follow-up period may have missed late-onset toxicities, potentially underestimating the long-term effect of treatment. Additionally, cosmetic outcomes were based on physician assessments rather than standardized patient-reported outcome measures, introducing a potential subjective bias. Furthermore, this study did not account for potential confounders that could influence these associations. Future studies with longer follow-up periods and validated patient-centered assessment tools are needed to confirm and expand these findings.

## 5 Conclusion

Ultra-hypofractionated RT using a 26 Gy in five fractions schedule was well tolerated in our patient population. A larger breast volume [in cc], higher BMI, and greater breast V105% and V107% of the prescribed dose were associated with increased toxicity. Optimizing modifiable dosimetric parameters may help to minimize treatment-related side effects. These findings provide important insights into the evolving role of ultra-hypofractionated regimens, particularly in diverse patient populations.

## Data Availability

The raw data supporting the conclusions of this article will be made available by the authors, without undue reservation.

## References

[1] Albeshan SM Alashban YI . Incidence trends of breast cancer in Saudi Arabia: a joinpoint regression analysis [2004–2016. J King Saud Univ Sci. (2021) 33. doi: 10.1016/j.jksus.2021.101578 38826717

[2] Alsolami FJ Azzeh FS Ghafouri KJ Ghaith MM Almaimani RA Almasmoum HA . Determinants of breast cancer in saudi women from makkah region: a case-control study (breast cancer risk factors among saudi women). BMC Public Health. (2019) 19:1554. doi: 10.1186/s12889-019-7942-3 31752790 PMC6873398

[3] Asiri S Asiri A Ulahannan S Alanazi M Humran A Hummadi A . Incidence rates of breast cancer by age and tumor characteristics among saudi women: recent trends. Cureus. (2020) 12:e6664. doi: 10.7759/cureus.6664 31966952 PMC6961791

[4] Rahman S Zayed H . Breast cancer in the gcc countries: a focus on brca1/2 and non-brca1/2 genes. Gene. (2018) 668:73–6. doi: 10.1016/j.gene.2018.05.045 29777908

[5] Darby S McGale P Correa C Taylor C Arriagada R Clarke M . Effect of radiotherapy after breast-conserving surgery on 10-year recurrence and 15-year breast cancer death: meta-analysis of individual patient data for 10,801 women in 17 randomised trials. Lancet. (2011) 378:1707–16. doi: 10.1016/S0140-6736(11)61629-2 PMC3254252 22019144

[6] Trabulsi NH Shabkah AA Ujaimi R Iskanderani O Kadi MS Aljabri N . Locally advanced breast cancer: treatment patterns and predictors of survival in a saudi tertiary center. Cureus. (2021) 13:e15526. doi: 10.7759/cureus.15526 34277162 PMC8269977

[7] Van de Steene J Vinh-Hung V Cutuli B Storme G . Adjuvant radiotherapy for breast cancer: effects of longer follow-up. Radiother Oncol. (2004) 72:35–43. doi: 10.1016/j.radonc.2004.04.004 15236872

[8] Budach W Bölke E Matuschek C . Hypofractionated radiotherapy as adjuvant treatment in early breast cancer. a review and meta-analysis of randomized controlled trials. Breast Care (Basel). (2015) 10:240–5. doi: 10.1159/000439007 26600759 PMC4608603

[9] Bentzen SM Agrawal RK Aird EG Barrett JM Barrett-Lee PJ Bliss JM . The uk standardization of breast radiotherapy (start) trial a of radiotherapy hypofractionation for treatment of early breast cancer: a randomized trial. Lancet Oncol. (2008) 9:331–41. doi: 10.1016/S1470-2045(08)70077-9 PMC2323709 18356109

[10] Bentzen SM Agrawal RK Aird EG Barrett JM Barrett-Lee PJ Bentzen SM . The uk standardization of breast radiotherapy (start) trial b of radiotherapy hypofractionation for treatment of early breast cancer: a randomized trial. Lancet. (2008) 371:1098–107. doi: 10.1016/S0140-6736(08)60348-7 PMC2277488 18355913

[11] Murray Brunt A Haviland JS Wheatley DA Sydenham MA Alhasso A Bloomfield DJ . Hypofractionated breast radiotherapy for 1 week versus 3 weeks (fast-forward): 5-year efficacy and late normal tissue effects results from a multicenter, non-inferiority, randomized, phase 3 trial. Lancet. (2020) 395:1613–26. doi: 10.1016/s0140-6736(20)30932-6 32580883 PMC7262592

[12] Brunt AM Haviland JS Wheatley DA Sydenham MA Bloomfield DJ Chan C . One versus three weeks hypofractionated whole breast radiotherapy for early breast cancer treatment: the fast-forward phase iii rct. Health Technol Assess. (2023) 27:1–176. doi: 10.3310/wwbf1044 37991196 PMC11017153

[13] Wang SL Fang H Song YW Wang WH Hu C Liu YP . Hypofractionated versus conventional fractionated postmastectomy radiotherapy for patients with high-risk breast cancer: a randomized, non-inferiority, open-label, phase 3 trial. Lancet Oncol. (2019) 20:352–60. doi: 10.1016/s1470-2045(18)30813-1 30711522

[14] Krug D Baumann R Combs SE Duma MN Dunst J Feyer P . Moderate hypofractionation remains the standard of care for whole-breast radiotherapy in breast cancer: considerations regarding fast and fast-forward. Strahlenther Onkol. (2021) 197:269–80. doi: 10.1007/s00066-020-01744-3 33507331 PMC7841378

[15] Arbab M Frame R Alluri P Parsons D Lin MH Cleaton J . Master breast radiation planning: simple guide for radiation oncology residents. Adv Radiat Oncol. (2024) 9. doi: 10.1016/j.adro.2024.101476 38690296 PMC11059315

[16] Society BP, Faculty of Pain Medicine . Outcome Measures. London, UK: British Pain Society (2019). Available online at: https://www.britishpainsociety.org/static/uploads/resources/files/Outcome_Measures_January_2019.pdf (Accessed April 19, 2026).

[17] Xie Y Wang Q Hu T Chen R Wang J Chang H . Risk factors related to acute radiation dermatitis in breast cancer patients after radiotherapy: a systematic review and meta-analysis. Front Oncol. (2021) 11:738851. doi: 10.3389/fonc.2021.738851 34912704 PMC8667470

[18] Ratosa I Jenko A Oblak I . Breast size impact on adjuvant radiotherapy adverse effects and dose parameters in treatment planning. Radiol Oncol. (2018) 52:233–44. doi: 10.2478/raon-2018-0026 30210048 PMC6137355

[19] Das IJ Cheng CW Fein DA Fowble B . Patterns of dose variability in radiation prescription of breast cancer. Radiother Oncol. (1997) 44:83–9. doi: 10.1016/s0167-8140(97)00054-6 9288862

[20] Haviland JS Owen JR Dewar JA Agrawal RK Barrett J Barrett-Lee PJ . The uk standardization of breast radiotherapy (start) trials of radiotherapy hypofractionation for treatment of early breast cancer: 10-year follow-up results of two randomized controlled trials. Lancet Oncol. (2013) 14:1086–94. doi: 10.1016/s1470-2045(13)70386-3 24055415

[21] Smith BD Bellon JR Blitzblau R Freedman G Haffty B Hahn C . Radiation therapy for the whole breast: executive summary of an american society for radiation oncology (astro) evidence-based guideline. Pract Radiat Oncol. (2018) 8:145–52. doi: 10.1016/j.prro.2018.01.012 29545124

[22] Patel AK Ling DC Richman AH Champ CE Huq MS Heron DE . Hypofractionated whole-breast irradiation in large-breasted women-is there a dosimetric predictor for acute skin toxicities? Int J Radiat Oncol Biol Phys. (2019) 103:71–7. doi: 10.1016/j.ijrobp.2018.08.024 30145393

[23] Sourial F Champ C Beriwal S . The critical role of dose homogeneity in modern breast hypofractionation. Int J Radiat Oncol Biol Phys. (2026) 116(2):241–50. doi: 10.1016/j.ijrobp.2026.01.005 41554414

[24] Mulla Z Taha H Ghandourh WA Hashem RM Alotaibi MA Habibullah HF . Impact of hypofractionated radiation protocols on reducing travel burden and improving patient satisfaction: when less is more. JCO Glob Oncol. (2025) 11:e2400488. doi: 10.1200/go-24-00488 40267380

[25] Rudat V Nour A Hammoud M Abou Ghaida S . Better compliance with hypofractionation vs. conventional fractionation in adjuvant breast cancer radiotherapy: results of a single, institutional, retrospective study. Strahlenther Onkol. (2017) 193:375–84. doi: 10.1007/s00066-017-1115-z 28233048 PMC5405099

[26] Patel S Olatunji E Mallum A Benjika BB Joseph AO Joseph S . Expanding radiotherapy access in sub-saharan africa: an analysis of travel burdens and patient-related benefits of hypofractionation. Front Oncol. (2023) 13:1136357. doi: 10.3389/fonc.2023.1136357 37143940 PMC10151787

[27] Yadav BS Dey T . Hypofractionation for regional nodal irradiation in breast cancer: best of both the worlds. Clin Breast Cancer. (2024) 24:399–410. doi: 10.1016/j.clbc.2024.03.007 38614852

[28] Piras A Menna S D’Aviero A Marazzi F Mazzini A Cussmano D . New fractionations in breast cancer: a dosimetric study of 3d-crt versus vmat. J Med Radiat Sci. (2021) 69:227–35. doi: 10.1002/jmrs.530 34551211 PMC9163458

