# Supplementary material for: Optimizing outcomes with less than more: multi-institutional experience of fast-forward fractionation and the impact of dosimetric parameters on toxicity in breast cancer patients
Source: Front Oncol. 2026 Jun 10;16:1861707. doi: 10.3389/fonc.2026.1861707 (PMC13290563; doi:10.3389/fonc.2026.1861707)
Supplement: Supplementary file 1 [file Table1.docx]

Study Variables and Endpoints:

This study evaluated the relationships between independent variables (predictors) and dependent variables (toxicity endpoints) to understand the impact of dosimetric and patient-related factors on treatment outcomes.

Dependent Variables (Toxicity Endpoints):

1. Breast Pain:
   - Patient-reported pain on a numerical scale ranging from 0 (no pain) to 10 (worst imaginable pain). Pain was categorized as significant if rated ≥5.
2. Breast Shrinkage:
   - Clinically assessed or patient-reported reduction in breast volume compared to the contralateral breast post-radiotherapy baseline.
3. Telangiectasia:
   - Presence of dilated superficial capillaries observed during clinical examination or reported by patients.
4. Breast Edema:
   - Swelling of the breast tissue evaluated during follow-up visits, based on clinical signs or patient complaints.
5. Breast Discomfort:
   - General discomfort or unease in the treated breast, encompassing sensations such as tightness or tenderness.
6. Skin Dermatitis:
   - Acute or chronic skin reactions classified according to the Radiation Therapy Oncology Group (RTOG) criteria:
     - Grade I: Mild erythema, dry desquamation.
     - Grade II: Moderate erythema, patchy moist desquamation in skin folds.
     - Grade III: Confluent moist desquamation or bleeding induced by minor trauma.
     - Grade IV: Skin necrosis or ulceration of full-thickness dermis; spontaneous bleeding requiring surgical intervention.
7. Breast Hardness (Fibrosis):
   - Palpable firmness or stiffness of the treated breast tissue, based on clinical signs or patient complaints post-radiotherapy baseline.
8. Hypo/Hyperpigmentation:
   - Visible discoloration of the skin in the treated area, categorized as either hypopigmentation (loss of skin color) or hyperpigmentation (darkening of skin).
9. Change in Breast Shape:
   - Patient-reported or clinically observed alteration in the contour or symmetry of the treated breast compared to the contralateral breast post-radiotherapy baseline.

Independent Variables (Predictors):

1. Breast Clinical Target Volume (CTV):
   - The volume of breast tissue identified as the target for radiotherapy, delineated based on imaging and clinical guidelines. Measured in cubic centimeters (cc).
2. Boost Dose:
   - Additional radiation dose administered to the tumor bed to enhance local control. Measured in Gray (Gy).
3. Plan’s Dmax:
   - The maximum dose delivered within the radiotherapy plan, expressed as an absolute dose (Gy).
4. Breast V107%:
   - The percentage of the breast volume receiving 107% or more of the prescribed dose.
5. Breast V105%:
   - The percentage of the breast volume receiving 105% or more of the prescribed dose, another metric for dose heterogeneity.
6. Breast Separation:
   - The distance between the anterior and posterior surfaces of the breast, measured at the central axis, indicating breast size and influencing dose distribution. Measured in centimeters (cm).
7. Body Mass Index (BMI):
   - A measure of body fat based on weight and height, calculated as weight in kilograms divided by height in meters squared (kg/m²).
8. Age:
   - Patient age at the time of diagnosis, recorded in years.
9. Neoadjuvant Chemotherapy:
   - Administration of systemic therapy (chemotherapy) prior to surgery and radiotherapy to shrink the tumor. Documented as a binary variable (Yes/No).
10. Adjuvant Chemotherapy:
    - Administration of systemic therapy (chemotherapy) after surgery and prior to radiotherapy to reduce recurrence risk. Documented as a binary variable (Yes/No).
11. Patient Comorbidities:
    - Presence of coexisting medical conditions (e.g., diabetes, hypertension, heart disease), recorded as categorical variables based on specific diagnoses.
